# Breast Reconstruction after Breast Implant-Associated Anaplastic Large Cell Lymphoma Treatment: A Case Report and Literature Review

**DOI:** 10.3390/jcm12051885

**Published:** 2023-02-27

**Authors:** Won-Seob Lee, Tae-Gon Kim, Jun-Ho Lee, Il-Kug Kim

**Affiliations:** Department of Plastic and Reconstructive Surgery, Yeungnam University College of Medicine, Daegu 42415, Republic of Korea

**Keywords:** BIA-ALCL, breast reconstruction, breast implants, silicone

## Abstract

Breast implant-associated anaplastic large cell lymphoma (BIA-ALCL) is a T-cell non-Hodgkin’s lymphoma that occurs in patients with at least one prior textured breast implant. BIA-ALCL has a relatively good prognosis when treated promptly. However, data on the methods and timing of the reconstruction process are lacking. Herein, we report the first case of BIA-ALCL in Republic of Korea in a patient who underwent breast reconstruction using implants and an acellular dermal matrix (ADM). A 47-year-old female patient was diagnosed with BIA-ALCL stage IIA (T4N0M0) and underwent bilateral breast augmentation using textured breast implants. She then underwent removal of both breast implants, total bilateral capsulectomy, adjuvant chemotherapy, and radiotherapy. There was no evidence of recurrence at 28 months postoperatively; therefore, the patient wished to undergo breast reconstruction surgery. A smooth surface implant was used to consider the patient’s desired breast volume and body mass index. The right breast was reconstructed with a smooth surface implant and an ADM in the prepectoral plane. Breast augmentation was performed on the left breast using a smooth surface implant. The patient was satisfied with the results and recovered fully with no complications.

## 1. Introduction

Breast implant-associated anaplastic large cell lymphoma (BIA-ALCL) is a T-cell non-Hodgkin’s lymphoma characterized as negative for both CD30 and anaplastic lymphoma kinase (ALK). BIA-ALCL occurs in patients with breast implants and those with at least one prior textured implant. The most common symptom is peri-implant fluid collection accompanied by breast swelling, pain, skin symptoms (e.g., inflammation and papules), and regional lymphadenopathy [1].

As of August 2021, 1078 patients were diagnosed with BIA-ALCL worldwide according to the Patient Registry and Outcomes for Breast Implants and Anaplastic Large Cell Lymphoma Etiology and Epidemiology (PROFILE) registry [2]. 

BIA-ALCL has been diagnosed in Asian patients, including Republic of Korea, Japan, and Thailand [3,4,5]. In Republic of Korea, textured breast implants were approved for use in 2007. Approximately 222,470 textured breast implants were implanted from 2007 to 2018. Histological and genetic characteristics of a case of BIA-ALCL were reported for the first time in 2019, and three patients have been diagnosed with BIA-ALCL to date in Republic of Korea [5,6,7].

BIA-ALCLs are primarily confined to the capsule and appear as confined peri-implant effusion or masses, in which neither capsular invasion nor systemic metastasis is common. Stage I can be treated with implant removal and surgery using en-bloc capsulectomy alone, whereas systemic therapies, such as chemotherapy, should be considered for BIA-ALCLs in stage II or higher [8,9]. Furthermore, BIA-ALCL has a relatively desirable prognosis [9] when diagnosed and treated promptly. 

Due to better understanding of BIA-ALCL and its desirable prognosis, breast reconstruction after treatment of BIA-ALCL is often considered [10]; however, data on the methods and timing of the reconstruction process remain limited [10]. Moreover, there are no reports of breast reconstruction post BIA-ALCL in Asia, including Republic of Korea. Herein, we report the first case of BIA-ALCL in Republic of Korea in a patient who underwent breast reconstruction using implants and an acellular dermal matrix (ADM), along with the treatment progress. We also reviewed relevant literature on the method and timing of reconstruction following BIA-ALCL treatment. 

## 2. Case Presentation 

### 2.1. Diagnosis and Treatment of BIA-ALCL

In July 2019, a 47-year-old female patient who underwent bilateral breast augmentation using silicone breast implants 7 years prior complained of swelling of the right breast. Ultrasound examination of the breast revealed fluid collections around the right implant. The right implant and surrounding fluid were removed, and a capsule biopsy was taken. The implant was identified as a BIOCELL silicone-filled textured breast implant (Allergan Inc., Irvine, CA, USA). Immunohistochemical analysis of the capsule tissue showed positive expression of CD3 and CD30 and negative expression of CD20 and anaplastic lymphoma kinase (ALK), leading to a diagnosis of BIA-ALCL. Positron emission tomography–computed tomography (PET–CT) showed no abnormal findings besides fluorodeoxyglucose uptake of the bilateral axilla. The patient underwent contralateral left breast implant removal, total bilateral capsulectomy of the breasts, and biopsies of the bilateral axillary lymph nodes. A biopsy identified infiltration of multiple lymphomas to the thickened peri-implant capsule, which, in the right axilla, is an extension of the right breast lymphoma. There was no metastasis in the left axilla, other lymph nodes, or distant organs. According to National Comprehensive Cancer Network guidelines, the patient was diagnosed with stage IIA (T4N0M0) lymphoma and underwent adjuvant chemotherapy and radiation therapy. Postoperatively, the patient underwent a follow-up observation with CT scans every 3 months. The patient showed no evidence of recurrence. 

### 2.2. Reconstruction after BIA-ALCL Treatment

Following treatment with BIA-ALCL, CT scans showed no evidence of recurrence. The patient was consulted regarding breast reconstruction 28 months post surgery. The patient strongly desired breast reconstruction and left breast augmentation. We thoroughly considered BIA-ALCL remission, desired breast volume, donor site morbidity, and feasibility of the reconstruction. The patient hoped for augmentation mammoplasty of the left breast using the previous volume, 280 mL. The patient’s body mass index (BMI) was approximately 20.99; therefore, it was inappropriate to use autologous tissue to obtain the desired breast volume for the patient on both sides. We planned to implement a right breast reconstruction and an augmentation of the left breast using a smooth surface implant.

The inframammary fold line and breast median line were marked using gentian violet to determine the place of breast implantation before surgery. During surgery, the incision was made according to the preoperative scar of the sub-nipple areolar area of the right breast. Dissection was performed in a prepectoral plane to create sufficient breast pockets for the insertion of the implant. A 240 mL implant (ES 261; Eurosilicone S.A.S, Apt, France) was used based on the volume of implants previously inserted and the volume of the ADMs to be used for reconstruction. The ADM, Megaderm (L&C Bio, Seoul, Republic of Korea), had a thickness of 1.0–1.5 mm and size of 18 × 18 cm^2^. The ADM was used to ensure a sufficient volume while wrapping the entire implant. Partial fixation to the pectoralis major muscle was performed to ensure correct implant placement within the breast pocket. The left breast underwent augmentation using the subfascial approach. A 280 mL implant (ES 261; Eurosilicone S.A.S, Apt, France) was used to create symmetry with the reconstructed right breast, and a wound drain was inserted on both sides. 

**Figure 1:**
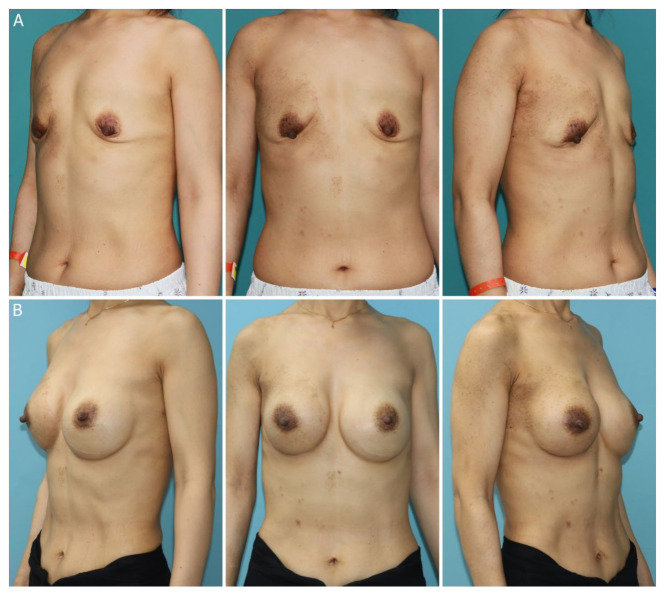
Pictures of the patient undergoing breast reconstruction following breast implant-associated anaplastic large cell lymphoma. (**A**) Left oblique view, anterior-posterior view, and right oblique of the patient prior to breast reconstruction. (**B**) Left oblique view, anterior-posterior view, and right oblique view of the patient 11 months post reconstruction.

**Figure 2:**
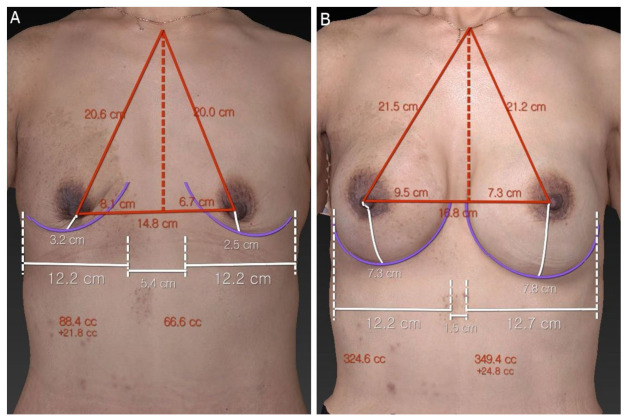
Three-dimensional image of the VECTRA Breast sculptor (Canfield Sci, Parsippany, NJ, USA). Evaluation with anatomical landmarks was performed on bilateral breasts. (**A**) Preoperative 3D image and (**B**) 11 months postoperative 3D image. The volume difference between the right breast (324.6 mL) and the left breast (349.4 mL) was 24.8 mL.

The patient had no postoperative complications, and both sutures and wound drains of the bilateral breasts were removed. The patient was discharged on day 13 postoperatively (Figure 1). There was no evidence of recurrence of BIA-ALCL based on chest and abdominal CT and breast ultrasound scans over 11 months postoperatively and 39 months after the first diagnosis of BIA-ALCL. Both surgical outcomes and patient satisfaction were evaluated based on VECTRA 3D imaging (Canfield Sci, Parsippany, NJ, USA) and the BREAST-Q Reconstruction Module at 11 months postoperatively (Figure 2). An evaluation of the VECTRA image based on the anatomic landmark confirmed that the bilateral breasts were symmetrically reconstructed. The volume difference between the right (324.6 mL) and left breasts (349.4 mL) was 24.8 mL. Postoperative patient satisfaction on the Likert scale regarding psychosocial well-being and aesthetic satisfaction was high at 11 months postoperatively. 

## 3. Discussion

Since Keech and Creech [11] first reported BIA-ALCL in 1997, several studies have analyzed the causes of BIA-ALCL. Textured breast implants have a strong relationship with BIA-ALCL [12,13,14,15]. Brody et al. [13] reviewed 173 BIA-ALCL cases, where all patients reported using at least one textured implant regardless of the manufacturer. However, the etiology and development of BIA-ALCL remain unclear. Some plausible hypotheses include immunological hypotheses, tribology, and subclinical infection [16,17,18]. These hypotheses commonly acknowledge that chronic inflammation induces tumorigenesis. 

BIA-ALCL has a substantially low recurrence rate following treatment. Surgically resectable T1 to T3 is 0%, and T4 is 14.3% [8]. This suggests that breast reconstruction can be considered in patients with BIA-ALCL who have undergone successful treatment. Longo et al. recommended reconstruction when there is no evidence of recurrence for more than 5 years based on the disease prognosis of disseminated peripheral T-cell lymphoma [19]. Since there was no evidence to completely preclude the correlation between smooth implant and BIA-ALCL, only reconstruction using autologous tissue was recommended. Clemens et al. indicated that replacement of smooth implants depends on patients’ preferences [20]. However, there have been limited studies on the type and timing of reconstruction. In a recent systematic review, information and analysis on breast reconstruction following BIA-ALCL were lacking [21]. Lamaris et al. [10] is the only study on the type and timing of reconstruction surgery. 

Lamaris et al. [10] retrospectively studied 18 patients treated for BIA-ALCL, followed by reconstruction. The authors found that IA-IC and BIA-ALCL staged IIA or higher accounted for 78% and 22% of the cases, respectively. Reconstruction was either performed immediately (7 cases) or delayed (11 cases). In total, 94% of patients were satisfied or very satisfied postoperatively. An algorithm was presented based on these findings; if the tumor is surgically resectable (IA-IC) and clearly confined within the capsule, an immediate reconstruction may be considered as a safe option. However, in cases where capsule confinement is not obvious, the authors recommended delaying reconstruction and considering other treatments, such as chemotherapy and repeat imaging (PET-CT), for 3–6 months. 

The patient in our case was diagnosed with IIA based on the identification of multiple lymphomas infiltrating the thickened peri-implant capsule and an extension of the breast lymphoma in the right axilla. The patient underwent chemotherapy and radiotherapy. There was no other meta-finding on CT for 2 years; this was to ensure the absence of recurrence after adjuvant therapy. Furthermore, reconstruction was performed appropriately using a smooth rather than textured implant. 

Eleven months after surgery, ongoing follow-ups showed no evidence of recurrence. Moreover, the patient was highly satisfied with the treatment of BIA-ALCL and the outcome of breast reconstruction surgery. 

Reconstruction methods after BIA-ALCL treatment include smooth implant, immediate mastopexy, autologous flap, and fat graft [10]. There is no guideline for determining the type of reconstruction. Autologous tissue transfer is regarded as the gold standard for breast reconstruction following mastectomy [22]. Autologous tissue transfer is a desirable option for patients who are dissatisfied with implants due to BIA-ALCL. However, in the present case, the patient hoped to have full-volume reconstruction and augmentation. As the patient’s BMI was approximately 20.99, it was difficult to secure a sufficient volume of autologous tissue, while the likelihood of donor site morbidity was high [23]. Therefore, we performed reconstruction and augmentation using an implant with a smooth surface after in-depth consultation with the patient.

According to the NCCN Clinical Practice Guidelines in Oncology (NCCN guidelines), 4.6% of patients with BIA-ALCL share the same pathology in the contralateral implants [9,24]. Therefore, it is important to periodically assess the opposite sides for BIA-ALCL. In the present case, there was no evidence of recurrence and metastasis based on follow-ups every 3 months over 2 years. 

In the present case, the recurrence and metastasis of BIA-ALCL were determined using CT. PET–CT imaging is recommended every 3 to 6 months if delayed reconstruction is considered [10]. Lamaris et al. only reported one case of recurrence in a patient with breast reconstruction following BIA-ALCL treatment. In this case of recurrence, the patient was diagnosed with BIA-ALCL stage IIA (T4N0M0) and treated with bilateral implant removal and capsulectomy. Breast reconstruction was performed with a bilateral DIEP flap 2 months post treatment. After 6 months of reconstruction, residual BIA-ALCL was found in the right breast and distant bone metastasis was also identified, emphasizing the need to fully assess the chance of recurrence following reconstruction surgery. However, in the previous case, treatment for BIA-ALCL was not appropriate, and the follow-up for delayed reconstruction was too short. Conversely, in the present case, we fully considered whether there should be a resection based on both the margin and associated mass at the time of surgery. Postoperatively, adjuvant therapies, such as chemotherapy and radiotherapy, were implemented in accordance with the guidelines. Moreover, we consulted with an oncologist to determine the presence of recurrence and metastasis in both CT and clinical examination results, rather than in PET–CT results alone. The patient was followed-up with over a long-term period of more than 2 years to confirm no recurrence or metastasis.

## 4. Conclusions

BIA-ALCL is a rare disease; hence, there have been limited cases of breast reconstruction in patients with BIA-ALCL. Moreover, there are no reports on the implementation of breast reconstruction in BIA-ALCL, particularly in Asia. This case report provides novel insights into both disease prognosis and post-treatment reconstruction surgery for BIA-ALCL cases.

## Data Availability

The data presented in this study are available on request from the corresponding author. The data are not publicly available due to consideration for the patients’ privacy.
